# Mucosal Lipocalin 2 Has Pro-Inflammatory and Iron-Sequestering Effects in Response to Bacterial Enterobactin

**DOI:** 10.1371/journal.ppat.1000622

**Published:** 2009-10-16

**Authors:** Michael A. Bachman, Virginia L. Miller, Jeffrey N. Weiser

**Affiliations:** 1 Department of Microbiology, University of Pennsylvania School of Medicine, Philadelphia, Pennsylvania, United States of America; 2 Department of Pathology, University of Pennsylvania School of Medicine, Philadelphia, Pennsylvania, United States of America; 3 Department of Genetics, The University of North Carolina, Chapel Hill, North Carolina, United States of America; 4 Department of Microbiology and Immunology, The University of North Carolina, Chapel Hill, North Carolina, United States of America; 5 Department of Pediatrics, University of Pennsylvania School of Medicine, Philadelphia, Pennsylvania, United States of America; Tufts University School of Medicine, United States of America

## Abstract

Nasal colonization by both gram-positive and gram-negative pathogens induces expression of the innate immune protein lipocalin 2 (Lcn2). Lcn2 binds and sequesters the iron-scavenging siderophore enterobactin (Ent), preventing bacterial iron acquisition. In addition, Lcn2 bound to Ent induces release of IL-8 from cultured respiratory cells. As a countermeasure, pathogens of the *Enterobacteriaceae* family such as *Klebsiella pneumoniae* produce additional siderophores such as yersiniabactin (Ybt) and contain the *iroA* locus encoding an Ent glycosylase that prevents Lcn2 binding. Whereas the ability of Lcn2 to sequester iron is well described, the ability of Lcn2 to induce inflammation during infection is unknown. To study each potential effect of Lcn2 on colonization, we exploited *K. pneumoniae* mutants that are predicted to be susceptible to Lcn2-mediated iron sequestration (*iroA ybtS* mutant) or inflammation (*iroA* mutant), or to not interact with Lcn2 (*entB* mutant). During murine nasal colonization, the *iroA ybtS* double mutant was inhibited in an Lcn2-dependent manner, indicating that the *iroA* locus protects against Lcn2-mediated growth inhibition. Since the *iroA* single mutant was not inhibited, production of Ybt circumvents the iron sequestration effect of Lcn2 binding to Ent. However, colonization with the *iroA* mutant induced an increased influx of neutrophils compared to the *entB* mutant. This enhanced neutrophil response to Ent-producing *K. pneumoniae* was Lcn2-dependent. These findings suggest that Lcn2 has both pro-inflammatory and iron-sequestering effects along the respiratory mucosa in response to bacterial Ent. Therefore, Lcn2 may represent a novel mechanism of sensing microbial metabolism to modulate the host response appropriately.

## Author Summary

Bacterial pathogens such as *Klebsiella pneumoniae* require iron and use secreted molecules called siderophores to strip iron from mammalian proteins. When bacteria colonize the upper respiratory tract, the mucosa secretes the protein lipocalin 2 (Lcn2) that binds to the siderophore enterobactin (Ent) and disrupts bacterial iron acquisition. In addition, Lcn2 bound to Ent stimulates release of the neutrophil-recruitment signal IL-8 from cultured respiratory cells. Some pathogens avoid Lcn2 binding by attaching glucose to Ent (to make Gly-Ent) or by making alternative siderophores. To determine the effect of Lcn2 on bacterial colonization, we colonized mice that express or lack Lcn2 with *K. pneumoniae* mutants that express or lack Ent, Gly-Ent and the alternative siderophore Yersiniabactin (Ybt). Our results indicate that mucosal Lcn2 inhibits colonization through iron sequestration and increases the influx of neutrophils in response to *K. pneumoniae* producing Ent. Therefore, Lcn2 acts as a barrier to colonization that pathogens must overcome to persist in the upper respiratory tract.

## Introduction

Bacteria utilize iron for electron transport, amino acid synthesis, DNA synthesis, and protection from superoxide radicals [1]. Under aerobic conditions, iron is primarily in the ferric (Fe (III)) oxidation state and readily forms insoluble complexes. Sequestration of the scarce quantities of soluble iron is a prototypical protective response against invading bacteria, mediated by the iron-binding proteins transferrin and lactoferrin and the storage protein ferritin. To acquire iron within the host, bacteria secrete siderophores such as enterobactin (Ent) that bind ferric iron with greater affinity than mammalian proteins.

In the thrust and parry between bacteria and their host to obtain iron, a new form of competition has been identified. Lipocalin 2 (Lcn2, also known as NGAL, siderocalin and 24p3) is a member of the lipocalin family of small-molecule transport proteins [2]. Lcn2 specifically binds Ent with an affinity similar to the *Escherichia coli* Ent receptor [2] and competes with bacteria for Ent binding.

Lcn2 is able to bind both ferric and aferric Ent, thereby depleting Ent from the microenvironment and inhibiting bacterial uptake of Ent-bound iron. As a result, Lcn2 is bacteriostatic. Bacterial growth can be restored by the addition of excess iron [2] or Ent [3]. In a murine sepsis model, serum Lcn2 is protective against an *E. coli* strain that requires Ent to obtain iron. Accordingly, Lcn2-deficient mice (*Lcn2^−/−^*) succumb to infection [3]. Conversely, co-injection of *E. coli* and a siderophore to which Lcn2 cannot bind causes lethal infection in *Lcn2^+/+^* mice [3].

Originally isolated from the specific granules of neutrophils, Lcn2 is also found in mucus producing cells of the respiratory tract [4]–[6]. In mice, the nasal mucosa responds to *Streptococcus pneumoniae* colonization by increasing Lcn2 mRNA expression (65-fold) [6]. Consequently, protein levels increase in olfactory glands and respiratory and olfactory epithelial cells. Lcn2 is secreted into the nasal lumen and bathes the colonized mucosa. Lcn2 is also induced by *Haemophilus influenzae* colonization, suggesting its production is a general response to colonizing bacteria [6].

In addition to sequestering iron, Lcn2 can act as a signaling molecule. A murine Lcn2 receptor, 24p3R, has been identified and is widely expressed in tissues including the lung and in lymphoid and myeloid cells [7]. In lymphocytic cells, 24p3R is able to internalize Lcn2 alone or Lcn2 bound to a siderophore. Internalization of Lcn2 bound to an iron-loaded siderophore increases the intracellular iron concentration. However, internalization of Lcn2 bound to an iron-free siderophore depletes intracellular iron levels by binding to cellular iron followed by export from the cell through recycling endosomes. In respiratory epithelial cells, Lcn2 elicits chemokine release [8]. In A549 and other human respiratory epithelial cell lines, incubation with aferric Ent produces a dose-dependent increase in the secretion of the chemokine IL-8 [8]. This response is potentiated by the addition of Lcn2 to form aferric Ent-Lcn2. In contrast, ferric Ent (Fe-Ent) does not elicit significant IL-8 release. Whether Lcn2 induces chemokine release and neutrophil recruitment during bacterial infection is unknown.

Perhaps due to the actions of Lcn2, successful pathogens do not typically depend solely on Ent for iron [9]. Pathogens such as *Klebsiella pneumoniae* often produce additional siderophores [9],[10] that Lcn2 is not predicted to bind [11]. *Salmonella enterica* and certain *K. pneumoniae* and *E. coli* isolates also encode the *iroA* locus that can counteract Lcn2 [12]–[17]. This cluster encodes the Ent glycosylase IroB that prevents Lcn2 binding, and IroC to export, IroN to import, and IroD to linearize glycosylated Ent [18],[19]. Transformation of *E. coli* with the *iroA* locus is sufficient to cause lethal infection in *Lcn2^+/+^* mice [19]. Conversely, disruption of either the *iroC* exporter or *iroB* glycoslyase attenuates virulence in a mouse model of systemic *Salmonella* infection [20]. In *K. pneumoniae*, this locus is associated with strains causing invasive disease (pyogenic liver abscesses) in patients [17].

Lcn2 could represent a new paradigm of mucosal immunity based on recognition of bacterial siderophores leading to direct iron sequestration and subsequent pro-inflammatory signaling. To test the antibacterial effects of mucosal Lcn2, a respiratory pathogen that produces Ent is required. A gram-negative, non-motile, encapsulated member of the family *Enterobacteriaceae*, *K. pneumoniae* colonizes both the nasopharynx and large intestine of humans [21], and is a common cause of bacterial pneumonia and sepsis. The wild-type *K. pneumoniae* strain ATCC 43816 produces Ent, contains the *iroA locus*, and produces a second siderophore, Yersiniabactin (Ybt) [22]. By exploiting defined *K. pneumoniae* siderophore mutants and Lcn2-deficient mice, we determined whether Lcn2 inhibits bacterial colonization by sequestering iron, promoting inflammation, or both.

## Results

### Establishment of a murine model of *K. pneumoniae* nasal colonization

**Figure 1:**
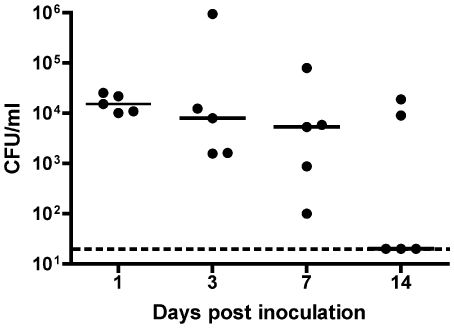
*K. pneumoniae* colonizes the nasopharynx of C57BL/6 mice. C57BL/6 mice were inoculated intranasally with 2×10^6^ cfu of an overnight LB culture of KPPR1 in 10 µL PBS. At each time point, 5 mice were sacrificed, nasopharyngeal lavage was performed, and lavage fluid was plated for quantitative culture. Colony forming unit (cfu) counts are shown as a scatter plot where the bar represents the median; the dashed line is the lower limit of detection (20 cfu/ml).

Previously a murine model of *K. pneumoniae* pneumonia was established and characterized [22],[23]. However, in order to test the role of Lcn2 in the upper respiratory tract we needed to develop a nasal colonization model. To do this, C57BL/6 mice were inoculated intranasally with increasing concentrations of *K. pneumoniae* in 10 µL of PBS. To facilitate recovery from the non-sterile nasopharynx, the strain KPPR1, a rifampin resistant-derivative of *K. pneumoniae* ATCC 43816 was used. Mice were sacrificed at day 1, 3, 7, or 14 and colonization was measured by tracheal cannulation and quantitative culture of nasopharyngeal lavage, as previously described [24]. Using 2×10^6^ cfu as the inoculum, sustained colonization at >5×10^3^ cfu/ml was observed for at least 7 days (Figure 1). Whereas instillation under anesthesia causes pneumonia [22],[23], inoculation of awake mice caused nasal carriage without systemic disease. *K. pneumoniae* was recovered only sporadically and at low density (<7×10^2^ cfu/organ) from the lungs and spleen (data not shown). Furthermore, all mice appeared healthy throughout the course of the experiment despite a 1000× larger inoculum compared to the pneumonia model.

**Figure 2:**
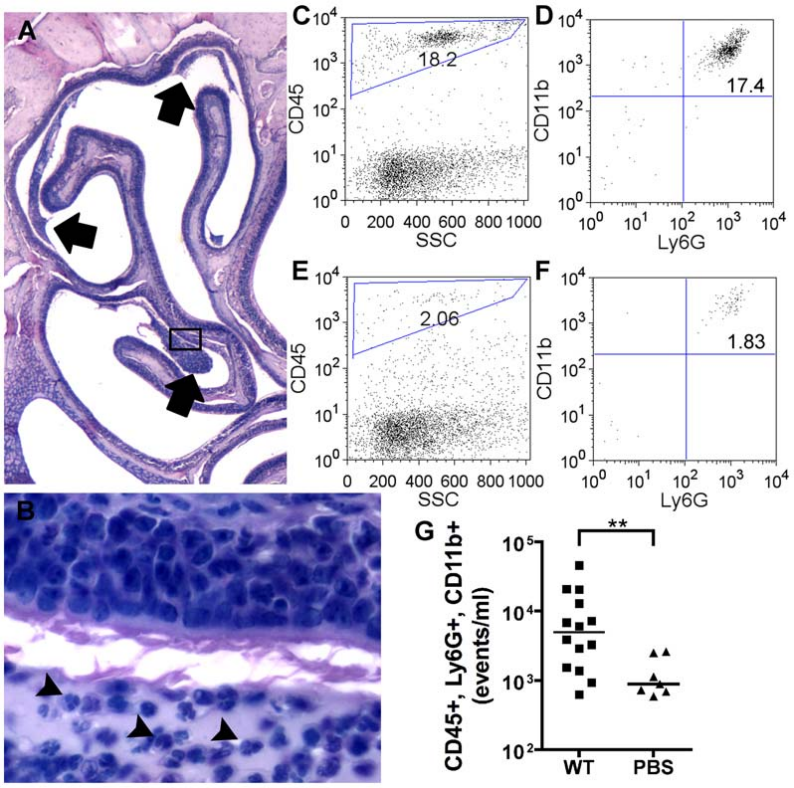
*K. pneumoniae* colonization elicits an acute inflammatory infiltrate. Mice colonized with wild-type *K. pneumoniae* KPPR1 were sacrificed at day 1 post-inoculation. Inflammatory infiltrates are seen adjacent to the turbinates of the nasopharynx (A, 20×, arrows) containing neutrophils (B, 600×, arrowheads). To enumerate the neutrophil influx, mice were sacrificed at day 3 post-inoculation for nasopharyngeal lavage, and 100 µL of lavage fluid was analyzed by flow cytometry. Neutrophils (CD45+, Ly6G+ and CD11b+) from a representative KPPR1 (C, D) or PBS mock-colonized (E, F) mouse lavage sample are shown. Compiled neutrophil counts are shown as a scatter plot; the bars represent median numbers of CD45+, Ly6G+, CD11b+ events/ml for each animal (G). p<0.01 by Mann-Whitney test.

To characterize the cellular response to *K. pneumoniae* nasal colonization, mice were inoculated as above and sacrificed at day 1 and day 3, skulls were dissected and decalcified, and saggital sections of the nasopharynx were examined by histology. On day 1, an acute inflammatory infiltrate in the paranasal airspaces was apparent in hematoxylin-eosin stained sections (Figure 2A). The lumen of the nasopharynx harbored dense clusters of neutrophils, as judged morphologically by high power light microscopy (Figure 2B). On day 3, an infiltrate of similar density and location was seen. No neutrophils were seen in the lumens of unchallenged mice (data not shown).

To directly quantify the neutrophil influx in response to *K. pneumoniae*, flow cytometry was performed on nasal washes of KPPR1-colonized and PBS mock-colonized mice. Activated neutrophils were identified by the following markers: CD45 (pan-hematopoietic marker), Ly6G (Granulocyte receptor-1) and CD11b (Mac-1). Aliquots of 100 µL/animal were stained with fluorophore-conjugated antibodies, and total numbers of neutrophils were compared (Figure 2C–F). On day 3, a marked increase in the percentage of total events that were CD45+, CD11b+, Ly6G+ neutrophils was seen in colonized versus uncolonized mice (Figure 2G, p<0.01).

### Neutrophils inhibit colonization and prevent bacteremia by wild-type *K. pneumoniae*

**Figure 3:**
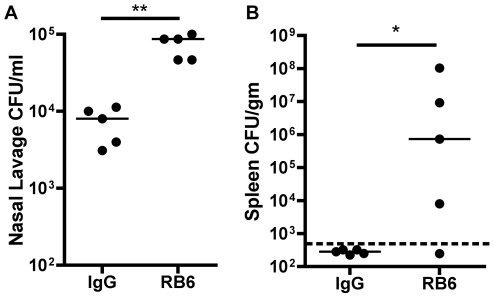
Neutrophils inhibit *K. pneumoniae* colonization and prevent bacteremia. Mice were treated with RB6-8C5 rat mAb to murine Ly6G or control rat IgG one day prior to inoculation with 2×10^6^ cfu of wild-type *K. pneumoniae* KPPR1 were sacrificed at day 1 post-inoculation. Nasopharyngeal lavage cfu/ml (A) and spleen cfu/gm (B) are shown as a scatter plot with the bar at the median; dashed line represents lower limit of detection. ** p<0.01 by Mann-Whitney test; * p<0.05 by Fisher's Exact Test for presence of detectable bacteria in the spleen.

To determine if neutrophils are protective during *K. pneumoniae* colonization, mice were depleted of neutrophils by the Ly6G-specific antibody RB6-8C5. One day prior to *K. pneumoniae* inoculation, mice were injected intraperitoneally with RB6-8C5 or control Rat IgG. Mice were inoculated intranasally with KPPR1 and subsequently sacrificed for nasopharyngeal lavage and quantitative spleen culture. At day 1 post-inoculation, RB6-8C5-treated mice had significantly greater colonization density compared to control-treated mice (p<0.01, Figure 3A). Four out of five RB6-8C5-treated mice were bacteremic, as judged by recovery of *K. pneumoniae* from the spleen, compared to none (0/5) of the control-treated mice (p<0.05, Figure 3B). By day 2, RB6-8C5-treated mice colonized with *K. pneumoniae* became moribund (data not shown). These data indicate that neutrophils control intranasal density of colonizing *K. pneumoniae* and prevent bacteremia and sepsis.

### Glycosylated-enterobactin or yersiniabactin are sufficient to support *K. pneumoniae* colonization

By exploiting isogenic siderophore mutants, *K. pneumoniae* can be used to evaluate two non-mutually exclusive functions of Lcn2: iron sequestration and pro-inflammatory activation. *K. pneumoniae* KPPR1 makes glycosylated Ent (Gly-Ent) and Ybt, neither of which Lcn2 is predicted to bind [11]. Constructing mutants in the *iroA* locus will allow study of the effects of Lcn2 binding to non-glycosylated Ent (Ent). However, whether Ybt can compensate for the loss of Ent bound by Lcn2 is unknown.

**Figure 4:**
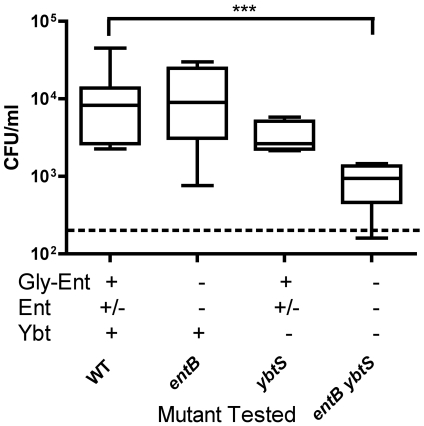
*K. pneumoniae* requires enterobactin or yersiniabactin, but not both, to colonize the nasopharynx efficiently. Colonization density was determined in C57BL/6 mice (n = 5 per group) at day 3 after intranasal inoculation with 2×10^6^ cfu of the *K. pneumoniae* mutants indicated. Box and whiskers graph shows the median and interquartile ranges; the dashed line is the lower limit of detection (20 cfu/ml). Siderophores encoded by each mutant are indicated by a plus (+). *** p<0.001 by Mann-Whitney test.

The role of Ybt and Gly-Ent in a mouse model of *K. pneumoniae* pneumonia was tested previously [22]. To determine the requirement of each siderophore during nasal colonization, cfu recovery of single and double mutants was compared to wild-type *K. pneumoniae* on day 3 post-inoculation (Figure 4). As determined by the density of *ybtS* and *entB* mutants, Gly-Ent or Ybt were sufficient to support *K. pneumoniae* nasal colonization. In contrast, the *entB ybtS* mutant strain lacking both siderophores was deficient in colonization (p<0.001 at day 3). These data indicate that siderophores are required for persistence on the nasal mucosa, but either Gly-Ent or Ybt is sufficient to scavenge the necessary iron. This is in contrast to the pneumonia model where the *ybtS* mutant had a distinct disadvantage compared to the *entB* mutant at later stages of infection [22].

### The *iroA* locus protects against lipocalin 2-mediated growth inhibition

To generate *K. pneumoniae* producing unmodified Ent that can be bound by Lcn2, the *iroA* locus was disrupted in the wild type and the *ybtS* mutant. The resulting *iroA ybtS* mutant is predicted to depend on unmodified Ent to acquire iron and therefore to be susceptible to Lcn2-mediated interference with iron acquisition. In contrast, the *iroA* mutant is predicted to make unmodified Ent, which may initiate Lcn2-mediated inflammation, but to be able to acquire iron using Ybt.

**Figure 5:**
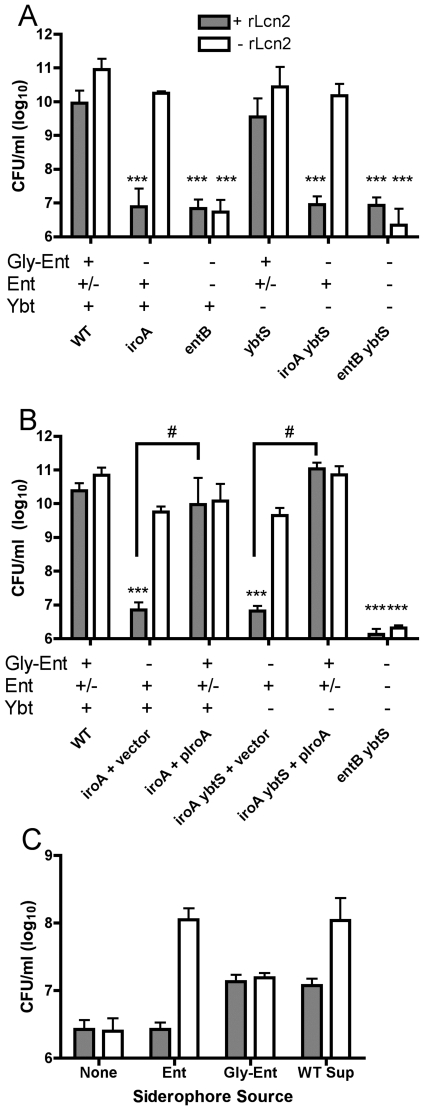
The *iroA* locus protects against growth inhibition by lipocalin 2. Overnight growth in Lcn2-deficient mouse serum with or without 1.6 µM recombinant Lcn2 was determined for the *K. pneumoniae* mutants indicated (A). For complementation studies, growth in serum was determined for the *iroA* and *iroA ybtS* mutant transformed with the vector control (pACYC184) or pIroA (pACYC184::*iroBCDN*) (B). Mean±SEM for at least three independent experiments is shown as log_10_ CFU/ml. Siderophores encoded by each mutant are indicated by a plus (+). *** p<0.001 comparing each condition to WT plus rLcn2, and # p<0.001 comparing pIroA to vector control, as determined by one-way ANOVA with Tukey's multiple comparison test. To determine if wild-type *K. pneumoniae* produces Lcn2-resistant and sensitive Ent, growth of the *entB* mutant in serum with or without 5 µM recombinant Lcn2 was compared between vehicle control, 16 nM of purified Ent or Gly-Ent (Salmochelin S4), or wild-type culture supernatant (1∶640 dilution) (C). Mean ± standard deviation for two independent experiments is shown as log_10_ CFU/ml.

To test the requirement of the *iroA* locus for growth in the presence of Lcn2, wild-type and siderophore mutant *K. pneumoniae* were grown in serum from *Lcn2^−/−^* mice with or without recombinant murine Lcn2 (rLcn2, 1.6 µM). Serum was chosen as a growth medium where Ent is required to obtain iron from transferrin [25],[26]. Production of either Ent or Gly-Ent was sufficient for maximal growth in *Lcn2^−/−^* serum (Figure 5, white bars). In contrast to colonization, Ybt was unable to support growth in mouse serum *in vitro* (see *entB* mutant). Addition of rLcn2 (shaded bars) inhibited Ent-producing *K. pneumoniae* (*iroA*, *iroA ybtS* mutants) by approximately 1000-fold compared to Gly-Ent-producing *K. pneumoniae* (wild type, *ybtS* mutant).

To confirm that Lcn2-sensitivity of the *iroA* and *iroA ybtS* mutants is attributable to disruption of the *iroA* locus, complementation studies were performed. The mutants contain a disruption in the initial gene *iroB* that may have polar effects on the rest of the operon. Transformation with plasmids containing *iroB* from *K. pneumoniae* KPPR1 or *E. coli χ*7122 was toxic and inhibited growth even in the absence of Lcn2 (data not shown). Therefore polar effects could not be ruled out. Instead, the mutants were transformed with a plasmid pIroA (pIJ137) encoding *iroBCDN* from *E. coli χ*7122. In *E. coli*, this construct confers the ability to produce Gly-Ent as determined by liquid chromatography-mass spectrometry [27]. Transformation with pIroA, but not the control plasmid, rescued growth of the *iroA* and *iroA ybtS* mutants in Lcn2-containing serum (Figure 5B). This indicates that the *iroA* locus is required to prevent Lcn2-dependent growth inhibition.

In *E. coli* and *Salmonella* strains encoding *iroA*, only a subset of enterobactin is glycosylated [20],[27]. Comparison of wild-type *K. pneumoniae* growth in the presence or absence of Lcn2 suggested that it produces a mixture of Lcn2-resistant and sensitive Ent (Figure 5A). To examine this possibility in more detail, culture supernatants from the wild type grown in iron-limited M9 broth were used to stimulate growth of the *entB* mutant in Lcn2-containing serum. As controls, purified Ent and Gly-Ent (Salmochelin S4) were used to stimulate growth. Gly-Ent supported identical levels of growth in the presence and absence of Lcn2, whereas Ent only stimulated growth in the absence of Lcn2. Culture supernatant from the wild type was partially sensitive to Lcn2, indicating that *K. pneumoniae* secretes a mixture of Lcn2-sensitive and resistant Ent (Figure 5C).

### The *iroA* locus is required for colonization by enterobactin-dependent *K. pneumoniae*

To test whether the *iroA* locus is required for bacterial colonization, C57BL/6 mice were inoculated with 2×10^6^ cfu of *K. pneumoniae* producing different combinations of Gly-Ent, Ent and Ybt. To enhance siderophore expression prior to inoculation, *K. pneumoniae* was grown in the presence of the iron chelator 2,2′-dipyridyl (200 µM). At this concentration of chelator, transcription of Ent-synthesis enzymes is increased ∼8 fold compared to rich media, based on a transcriptional GFP fusion to *entC* (data not shown).

**Figure 6:**
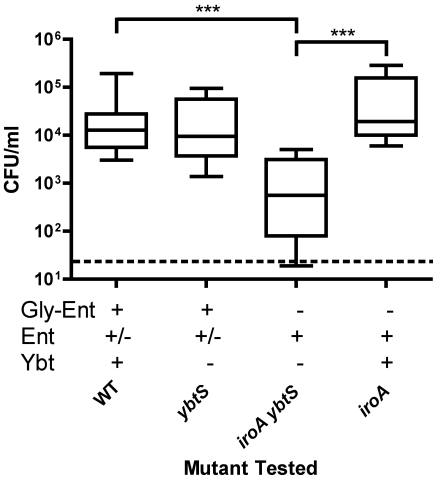
Enterobactin-dependent *K. pneumoniae* is defective in colonization. Colonization density at day 3 after intranasal inoculation with 2×10^6^ cfu of the *K. pneumoniae* mutants indicated was determined in C57BL/6 mice (n≥5 mice per group). Box and whiskers graph shows the median and interquartile ranges; the dashed line is the lower limit of detection (20 cfu/ml). Siderophores encoded by each mutant are indicated by a plus (+). *** p<0.001 as determined by Kruskall-Wallis Test with Dunn's post test.

Gly-Ent-producing *K. pneumoniae* colonized efficiently with or without producing Ybt (Figure 6, compare wild type and *ybtS*). In contrast, *K. pneumoniae* producing only Ent (*iroA ybtS* mutant) was deficient for colonization (p<0.001 at day 3). This defect in colonization could be due to a defect in iron acquisition, or through additional host defenses including pro-inflammatory responses triggered by Ent-Lcn2 signaling. As shown above, Ybt is able to support colonization in the absence of Ent (Figure 4). To test for defects in colonization independent from iron acquisition, carriage of *K. pneumoniae* making Ent plus Ybt (*iroA* mutant) was examined. The ability of the *iroA* mutant to produce Ybt restored maximal colonization. These data indicate that iron sequestration is an important host mechanism for inhibiting colonization of Ent-dependent *K. pneumoniae*.

### Mucosal lipocalin 2 inhibits colonization of *K. pneumoniae* dependent on unmodified enterobactin

**Figure 7:**
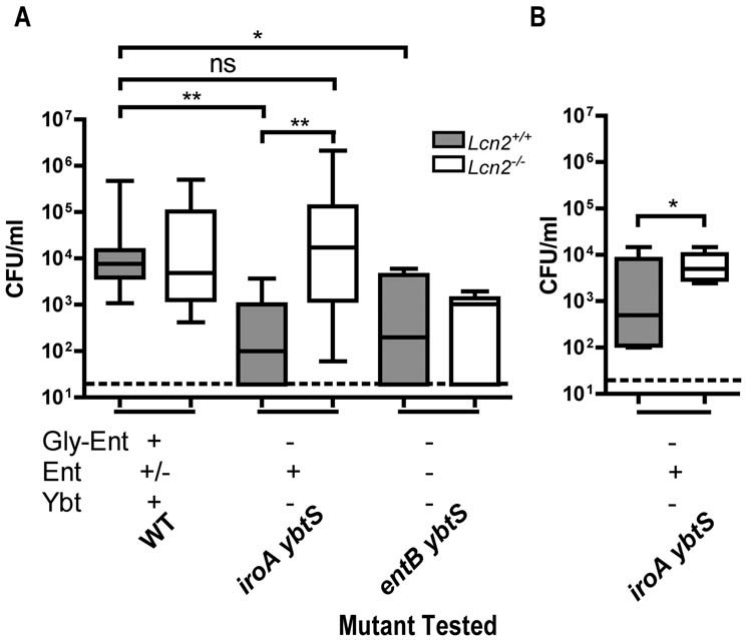
Mucosal lipocalin 2 is required to inhibit colonization by enterobactin-dependent *K. pneumoniae*. Colonization density of *Lcn2^+/+^* (shaded symbols) and *Lcn2^−/−^* mice (open symbols) on day 3 after intranasal inoculation with 2×10^6^ cfu of the *K. pneumoniae* mutants indicated (A). Box and whiskers graph shows the median and interquartile ranges for ≥10 mice per group; the dashed line is the lower limit of detection (20 cfu/ml). Siderophores encoded by each mutant are indicated by a plus (+). * p<0.05, ** p<0.01, ^ns^ p>0.05 as determined by Kruskall-Wallis Test with Dunn's post test. To remove the contribution of neutrophil-produced Lcn2, colonization density on day 1 was determined in *Lcn2^+/+^* (n = 6) and *Lcn2^−/−^* mice (n = 8) treated with RB6-8C5 rat mAb to murine Ly6G one day prior to inoculation with 2×10^6^ cfu of *iroA ybtS* mutant *K. pneumoniae* (B). * p<0.05 as determined by Mann-Whitney Test.

To determine whether Lcn2 is required to inhibit Ent-dependent bacteria, colonization of Lcn2-producing (*Lcn2^+/+^*) and Lcn2-deficient littermate mice (*Lcn2^−/−^*) was compared (Figure 7A). Wild-type *K. pneumoniae* producing Gly-Ent and Ybt maximally colonized both *Lcn2^+/+^* and *Lcn2^−/−^* mice. Conversely, the *iroA ybtS* mutant *K. pneumoniae* dependent on Ent was significantly inhibited in *Lcn2^+/+^* mice (p<0.01). This inhibition was absent in *Lcn2^−/−^* mice and was similar to the defect seen in the *entB ybtS* mutant. Therefore, Lcn2 is required to inhibit Ent-dependent strains and acts predominantly by interfering with iron acquisition.

Two potential sources of Lcn2 in the nasal lumen are the mucosa and infiltrating neutrophils. To determine whether mucosal Lcn2 is able to inhibit Ent-dependent *K. pneumoniae* colonization, mice were depleted of neutrophils using the RB6-8C5 antibody one day prior to inoculation. Despite RB6-8C5 treatment, colonization was significantly inhibited in *Lcn2^+/+^* compared to *Lcn2^−/−^* mice at day 1 (p<0.05, Figure 7B). These data indicate that mucosal sources of Lcn2 are sufficient to provide its antibacterial effects during colonization.

### Lipocalin 2 induces IL-8 release from cultured respiratory cells in response to unmodified but not glycosylated enterobactin

**Figure 8:**
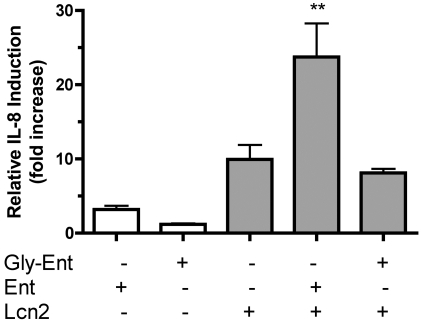
Lipocalin 2 does not stimulate IL-8 release from A549 respiratory cells in response to glycosylated enterobactin. IL-8 release from cultured A549 respiratory cells was measured by ELISA after overnight stimulation with 50 µM Ent or Gly-Ent (Salmochelin S4) and/or 25 µM recombinant Lcn2 (shaded bars). Mean±SEM of fold-increase above vehicle control from at least three independent experiments is shown. ** p<0.01 comparing Ent-Lcn2 to all other stimuli as determined by one-way ANOVA with Tukey's multiple comparison test.

Although iron sequestration is the predominant effect observed during colonization, Lcn2 may have additional pro-inflammatory effects that are blocked by glycosylation of Ent. In cultured respiratory cells, the combination of Lcn2 and aferric Ent induces synergistic IL-8 release [8]. To determine whether glycosylation of Ent prevents this synergistic activation of chemokine release, A549 human respiratory cells were incubated with combinations of purified Ent, Gly-Ent, or rLcn2. Whereas co-incubation of Ent with Lcn2 induced IL-8 release greater than 20-fold, co-incubation of Gly-Ent and Lcn2 did not stimulate IL-8 release above the level induced by Lcn2 alone (Figure 8). No significant cellular cytotoxicity, as measured by LDH release, was detected for any combination of stimuli (data not shown). Consistent with the observation that expression of the *iroA* locus prevents Lcn2 binding, Lcn2 does not stimulate IL-8 release from cultured respiratory cells in response to Gly-Ent.

### Enterobactin-producing *K. pneumoniae* elicit a lipocalin 2-dependent increase in neutrophil influx

**Figure 9:**
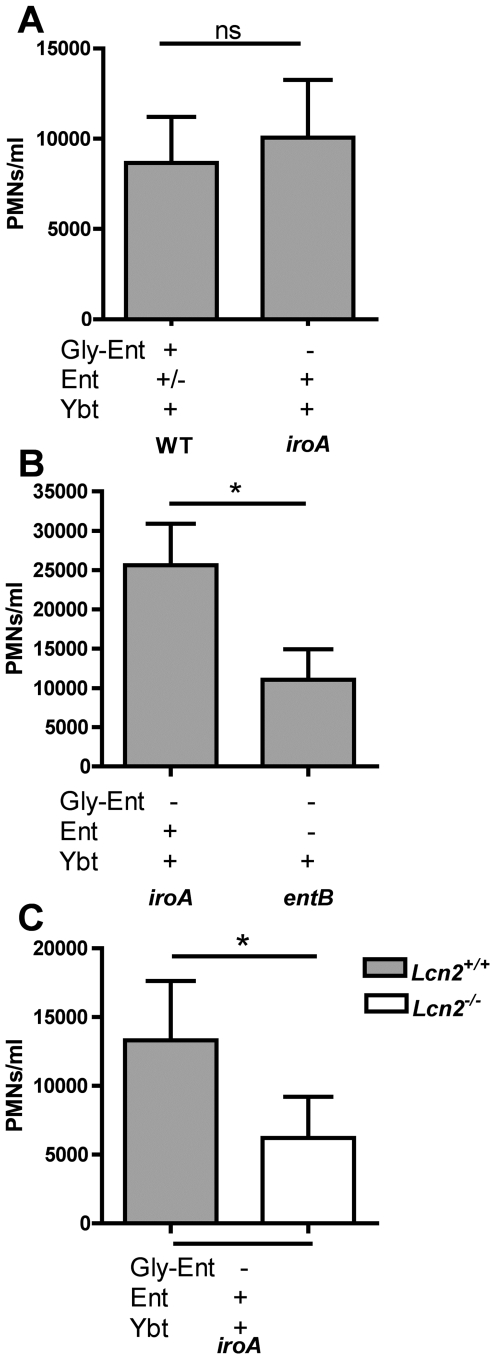
Lipocalin 2 promotes increased neutrophil influx in response to enterobactin-producing *K. pneumoniae*. Intraluminal neutrophil counts (PMNs) were measured by flow cytometry of 100 µL nasopharyngeal lavage fluid on day 3 after intranasal inoculation of C57BL/6 mice with 2×10^6^ cfu of the following combinations of *K. pneumoniae*: wild-type KPPR1 vs. *iroA* mutant (A), or *iroA* mutant vs. *entB* mutant (B). *p<0.05 by the Mann-Whitney test. To determine if neutrophil influx to Ent-producing strains is Lcn2-dependent, *Lcn2^+/+^* and *Lcn2^−/−^* littermates were colonized with 2×10^6^ cfu of *iroA* mutant *K. pneumoniae* (C). * p<0.05 as determined by the Wilcoxon matched pair test. Data points represent CD45+, Ly6G+, CD11b+ events and dotted lines connect littermates (n≥5 per group). Siderophores encoded by each mutant are indicated by a plus (+).

The induction of the neutrophil chemoattractant IL-8 *in vitro* suggests that Ent release by colonizing bacteria may stimulate neutrophil recruitment during infection. To measure the effect of Ent on neutrophil influx, flow cytometry was performed on nasal lavage fluid from mice colonized with wild type, *iroA* and *entB* mutants. The *iroA* mutant is predicted to produce Ent that interacts with Lcn2, is not predicted to be susceptible to iron sequestration since it makes Ybt, and colonizes similarly to the wild type (Figure 7). In contrast, the *entB* mutant produces no Ent but also colonizes similarly to the wild type (Figure 4). Comparison of the wild type and *iroA* mutant demonstrated no difference in neutrophil influx (Figure 9A). This suggests that the subset of unmodified Ent produced by the wild type is sufficient to induce a robust inflammatory response. To measure cellular inflammation directly attributable to unmodified Ent, neutrophil counts were compared between *iroA* and *entB*-colonized mice. The *iroA* and *entB* mutants achieved a similar level of colonization (data not shown), but the *iroA* mutant elicited significantly more neutrophils (Figure 9B). To determine if the neutrophil influx in response to Ent-producing *Klebsiella* is Lcn2-dependent, *Lcn2^+/+^* and *Lcn2^−/−^* littermates were colonized with *iroA* mutant *K. pneumoniae*. *Lcn2^+/+^* mice had a significantly greater neutrophil influx than *Lcn2^−/−^* littermates in response to the Ent-producing *iroA* mutant (Figure 9C, p<0.05 Wilcoxon matched pair test). The lower number of neutrophils in *Lcn2^−/−^* mice could not be attributed to decreased colonization density (data not shown). Concurrent with its predominant iron sequestration effect, Lcn2 also appears to induce greater neutrophil influx in response to bacteria producing unmodified Ent.

## Discussion

This work establishes an animal model to study the function of Lcn2 in the upper respiratory tract. As well as being an important human pathogen, *K. pneumoniae* is a tractable model organism that colonizes the nasopharynx and can be genetically manipulated to produce siderophore mutants that do or do not interact with Lcn2. Colonization with wild-type *K. pneumoniae* persists for at least seven days, and induces a robust acute inflammatory response. The fact that intranasal inoculation of awake mice produces colonization, and not pneumonia, is likely because the mice do not aspirate nasopharyngeal contents into their lungs [28].

Similarly to pneumonia, persistence of *K. pneumoniae* in the upper respiratory tract requires ongoing iron acquisition [22]. Either Gly-Ent or Ybt are required for maximal colonization. This confirms that the nasal mucosa is an iron-limited environment for bacteria, presumably due to the high lactoferrin concentration in nasal secretions [29],[30]. Furthermore, this suggests that colonization by *K. pneumoniae* requires bacterial replication, and not simply persistence of the originally inoculated organisms. Having established the need for *K. pneumoniae* to acquire iron in the nasopharynx, this model can be exploited to examine how host Lcn2 and the bacterial *iroA* locus affect the level of colonization.

By using *Lcn2^−/−^* mice and *K. pneumoniae* siderophore mutants, this study demonstrates that Lcn2 inhibits nasopharyngeal colonization by bacteria producing unmodified Ent. Although a seemingly narrow target for antimicrobial activity, Ent is produced by many members of the gram-negative *Enterobacteriaceae* family [1]. Therefore, Lcn2 may contribute to the tropism of enteric commensals for the gut rather than the respiratory tract. The respiratory tract secretes Lcn2 at basal levels and rapidly upregulates Lcn2 expression during colonization [4],[6]. In contrast, the large intestine does not express basal Lcn2 despite the presence of huge numbers of colonizing bacteria [4]. However, *Salmonella* infection induces Lcn2 production in the intestine, and the ability to utilize Gly-Ent confers a competitive advantage over an *iroN* mutant during intestinal inflammation [31]. Therefore, pathogenic *Enterobacteriaceae* such as *Salmonella* and *K. pneumoniae* can use Gly-Ent to obtain iron in an otherwise restricted environment.

In the respiratory tract, Lcn2 may provide selective pressure for Ent-independent methods of iron acquisition. Many clinical isolates of *K. pneumoniae* produce either aerobactin or Ybt [9],[10] in addition to Ent. Lcn2 binds bacillibactin of *Bacillus anthracis*, but *B. anthracis* also produces the unusual siderophore petrobactin that Lcn2 cannot bind [32]. Likewise, pathogens such as *S. pneumoniae* and *H. influenzae* appear to acquire iron in the respiratory tract without producing siderophores [33],[34].

In the pathogenic *K. pneumoniae* strain used here, either Gly-Ent or Ybt can support colonization of the nasopharynx. However, Ybt cannot support growth in serum. This defect could be due to an inability of Ybt to strip iron off of transferrin, or a serum component other than Lcn2 that inhibits Ybt-mediated iron acquisition. In contrast, the *iroA* locus supports robust growth in the presence of Lcn2. Therefore, Ybt and Gly-Ent are not functionally redundant in *K. pneumoniae*, and likely reflect adaptation to growth in the disparate environments of the respiratory, urinary, and intestinal tracts and the bloodstream.

*K. pneumoniae* nasal colonization induces a robust inflammatory response characterized by a rapid influx of neutrophils. Neutrophils limit colonization of wild-type *K. pneumoniae* and prevent hematogenous spread to the spleen. Despite producing Lcn2, neutrophils likely inhibit *K. pneumoniae* by predominantly Lcn2-independent mechanisms based on the following observations. In the presence of neutrophils, colonization by wild-type *K. pneumoniae* is the same in *Lcn2^+/+^* and *Lcn2^−/−^* mice. In contrast, depletion of neutrophils causes a 10-fold increase in wild-type colonization. Finally, mucosal Lcn2 is able to inhibit colonization of *iroA ybtS K. pneumoniae* despite depletion of neutrophils. Together, these data indicate that iron sequestration by mucosal Lcn2 is complementary to the antimicrobial actions of neutrophils.

Lcn2 also appears to induce a pro-inflammatory response from respiratory cells when bound to Ent. *In vitro*, Ent combined with Lcn2 causes a synergistic release of IL-8 from human respiratory cells, but Gly-Ent combined with Lcn2 does not. IL-8 is a neutrophil attracting chemokine, suggesting that signaling by Ent-Lcn2 leads to the recruitment of neutrophils. Accordingly, Ent-producing *K. pneumoniae* induce nasopharyngeal neutrophil influx in an Lcn2-dependent manner.

The potential signaling pathway between Lcn2, Ent and neutrophil recruitment is unknown. There is no direct IL-8 (CXCL-8) homologue in the mouse [35],[36], although there are several CXC chemokines such as Mip-2, KC, and LIX that have been shown to be induced by *K. pneumoniae* respiratory infections [37]. Studies to determine the effects of Ent and Lcn2 on murine CXC chemokine production from the murine respiratory mucosa are underway.

The data from this study suggest the following model in which Lcn2 monitors iron utilization by bacteria and activates the immune response when iron stores become depleted: At low bacterial density, secreted Ent binds Fe and Lcn2 in turn binds Fe-Ent to prevent delivery of iron to bacteria. The Fe-Ent-Lcn2 complex is internalized by respiratory epithelial cells [8], and could serve as both a signal of controlled colonization and a mechanism of iron recycling. We hypothesize that as bacterial density increases Lcn2 becomes a pro-inflammatory signal. When bacterial growth outpaces Fe availability, an increased proportion of Ent will be aferric. This aferric Ent-Lcn2 complex could be internalized and serve as a signal of uncontrolled bacterial replication to the respiratory epithelium. *In vitro*, respiratory cells respond to Ent-Lcn2 by induction of chemokines, an effect that could explain the increased neutrophil influx seen in response to Ent-producing *K. pneumoniae in vivo*.

The combined data from neutrophil and bacterial counts are consistent with the hypothesis that Lcn2 has a continuum of iron-sequestering and pro-inflammatory activities. Specifically, Ent-producing *K. pneumoniae* elicit neutrophils in *Lcn2^+/+^* mice without showing a defect in colonization. The bacterial density of colonization by *K. pneumoniae* is relatively low (∼1e^4^ CFU/ml) compared to counts from the pneumonia model [23]. If Fe is not depleted during colonization, then Lcn2 may bind primarily Fe-Ent. The small percentage of aferric Ent-Lcn2 could be sufficient to produce a modest increase in the number of neutrophils elicited by *K. pneumoniae* colonization. Whereas total depletion of neutrophils leads to increased bacterial numbers (Figure 3), this incremental increase in neutrophils is not sufficient to affect the density of colonizing organisms. If a perturbance in the microenvironment caused a large increase in bacterial levels, we would predict a greater pro-inflammatory response elicited by Ent-Lcn2. Alternatively, if *K. pneumoniae* reaches the lower respiratory tract where it can replicate to high numbers (>10^9^ CFU/gm) [22], there may be a dramatic increase in Ent-Lcn2 formation with a more significant effect of Lcn2 on the immune response. Consistent with this model, Chan and colleagues report that Lcn2 limits growth of *K. pneumoniae* ATCC 43816 (the parent strain of our wild type KPPR1) during pneumonia [38]. Since Ent is dispensable for growth during pneumonia from KPPR1 [22], iron sequestration by Lcn2 cannot account for the observed growth inhibition. This suggests that Lcn2 controls bacterial growth by an additional mechanism such as neutrophil recruitment. Whether Ent production stimulates Lcn2-dependent inflammation during pneumonia remains to be determined. Studying the pro-inflammatory effects of Lcn2 on the mucosal surface could reveal a new paradigm of innate immune signaling in response to bacterial metabolism.

## Materials and Methods

### Bacterial strains and media

KPPR1, a rifampin-resistant derivative of *K. pneumoniae* subsp. *pneumoniae* (ATCC 43816), with a type 1 O antigen and type 2 capsule was used as the wild-type strain in these studies [23]. Measurement of *entC* expression was performed using KPPR1 harboring the *entC* promoter-GFP reporter plasmid pE2 (VK096) [22]. Siderophore mutant strains of KPPR1 contain in-frame deletions of the enterobactin synthesis gene *entB* encoding 2,3-dihydro-2,3 dihydroxybenzoate synthase (VK087), the yersiniabactin synthesis gene *ybtS* encoding salicylate synthase (VK088) or both (VK089) [22]. Unless otherwise noted, strains were grown overnight in Luria-Bertani (LB) media, either at 30°C on agar or at 37°C shaking in broth. Media was supplemented with rifampin (30 µg/ml) or kanamycin (50 µg/ml) as needed. To stimulate siderophore production strains were grown overnight in LB broth supplemented with 200 µM 2,2′-dipyridyl (Acros Organics, Geel, Belgium), back diluted to OD_600_ 0.1, and grown an additional two hours to mid-logarithmic phase.

### *iroA* mutant construction

PCR primers specific for conserved regions of the *iroB* glycosylase gene (iroBfor 5′-GTGATGCAAACCGTCGGCTTC, iroBrev 5′-ACCATCGGTTTGACGGTGCCGAG) were constructed by comparing DNA sequences from *Salmonella typhimurium* LT2, *E. coli* CFT073, *E. coli* UT189, and *K. pneumoniae* virulence plasmid pLVPK. An internal 0.3 kb *iroB* PCR fragment was amplified, sequenced, and found to be 96% identical to *iroB* from pLVPK. This fragment was then cloned into the TA-based PCR cloning vector pCR2.1 (Invitrogen, Carlsbad, CA) and transferred to a kanamycin-resistant derivative of the λ*pir*-dependent suicide vector pGP704 [39]. This *iroB* suicide vector was transformed into *E. coli* strain BW20767 (ATCC 47084, RP4-2tet::Mu-1kan::Tn7-integrant *uidA*(ΔMlu1)::*pir+ recA1 creB510 leu-63 hsdR17 endA1 zbf-5 thi*) and subsequently conjugated into wild-type (KPPRI) and *ybtS* mutant (VK088) *K. pneumoniae* to generate *iroA* and *iroA ybtS* mutants. Integration of the suicide vector into the *iroB* gene was confirmed by generation of a novel PCR product using one primer on the vector (pGP704 MCS Pst>Xba 5′-GGTCGACGGATCCCAAG) and an *iroB* primer 5′ of the insertion site (iroBORF for 5′ ATGCGTATTTTATTTATAGGTCC). For complementation studies, each mutant was transformed by electroporation with either pACYC184::*iroB* (pIJ53) or pACYC184::*iroBCDN* (pIJ137, referred to as pIroA in this study) containing *iroA* genes from *E. coli* χ7122 [27]. The vector pACYC184 was transformed as a negative control.

### Nasal colonization model

All animals were handled in strict accordance with good animal practice as defined by the relevant national and/or local animal welfare bodies, and all animal work was approved by the University of Pennsylvania Institutional Animal Care and Use Committee (Assurance # A3079-01). For nasal colonization experiments, six to eight week-old C57BL/6 (Jackson Labs, Jackson, ME) mice were atraumatically inoculated intranasally without anesthesia with 2×10^6^ cfu of *K. pneumoniae*. LB broth grown cultures were centrifuged, resuspended in phosphate buffered saline (PBS), and 10 µL of the suspension was applied equally to both nares. To determine colonization density, mice were sacrificed by CO_2_ asphyxiation, the trachea was exposed and cannulated, 200 µL PBS was instilled, and lavage fluid was collected from the nares. Aliquots of lavage fluid were plated on LB agar supplemented with rifampin with a lower limit of detection of 20 cfu/ml. To measure lung or spleen infection, the organ was removed, weighed, homogenized in 500 µL PBS, plated on LB agar supplemented with rifampin, and quantified as cfu/gm. To examine the infection by histology, skulls were removed by decapitation, fixed by 48 h incubation in 10% neutral buffered formalin (Sigma-Aldrich, St. Louis, MO), and decalcified for 48 h in Cal-EX decalcifying solution (Fisher Scientific, Fair Lawn, NJ). Saggital sections of the nasal cavity were paraffin embedded and processed for hematoxylin and eosin (H&E) staining.

To deplete neutrophils, 145 µg of the rat anti-mouse IgG2B mAb RB6-BC5 directed against Ly-6G on the surface of mouse granulocytes was injected intraperitoneally 24 h prior to intranasal bacterial inoculation. This dose has been shown previously to result in peripheral blood neutropenia for at least 96 h (<50 granulocytes/ml, [6]). Intraperitoneal injection of 145 µg of rat total IgG was used as a control.

### Lipocalin 2-deficient mice

To ensure similar endogenous nasal flora, *Lcn2^−/−^* mice [3] (provided by Shizuo Akira via Alan Aderem) and *Lcn2^+/+^* littermates were compared. Offspring from heterozygous breeding pairs were genotyped using DNA extracted from tail samples with the DNeasy Blood and Tissue Kit (Qiagen, Germantown, MD). For each mouse, paired PCR amplifications were performed with a common primer (common 5′-CACATCTCATGCTGCTGAGATAGCCAC) and a primer specific for either the intact *Lcn2* locus (wild-type 5′-GTCCCTCTCACTTTGACAGAAGTCAGG) or the neomycin-cassette disrupted *Lcn2* locus (neo1500 5′-ATCGCCTTCTATCGCCTTCTTGACGAG).

### Recombinant lipocalin 2 production

Recombinant mouse lipocalin 2 (rLcn2) was purified as previously described [40]. Briefly, *E. coli* strain BL-21 expressing plasmid-encoded mouse lipocalin 2 as a glutathione *S*-transferase fusion protein (a gift from J. Barasch) was grown to mid-logarithmic phase in Terrific Broth supplemented with 50 µM ferrous sulfate and induced with 0.2 mM IPTG. Cells were harvested and lysed by sonication, and rLcn2 was purified on a Glutathione Sepharose 4B bead column (GE Amersham, Piscataway, NJ) followed by digestion with human thrombin (Sigma-Aldrich, St. Louis, MO) and elution in PBS. Purified rLcn2 was quantified using the Micro BCA Protein Assay Kit (Pierce, Rockford, IL) and siderophore-binding activity was confirmed by incubation with Fe-Ent followed by measurement of absorbance at 340 nm.

### Serum growth assay

Bacterial growth using mouse serum as an iron source was assayed as previously described [3]. RPMI supplemented with 10% heat-inactivated *Lcn2^−/−^* -mouse serum was inoculated with 1×10^3^ cfu/ml of an overnight culture of *K. pneumoniae* and incubated an additional 24 h at 37°C with 5% CO_2_ in 96-well plates. Bacterial growth was quantified by plating serial dilutions on LB agar. Where indicated, RPMI was supplemented with 1.6 µM recombinant Lcn2. To determine if the wild type produces Lcn2-resistant and sensitive Ent, sterile-filtered culture supernatants were collected from wild-type KPPR1 grown overnight in iron-limited M9 minimal media. RPMI supplemented with 3% heat-inactivated mouse serum was inoculated with 5×10^3^ cfu/ml of an overnight culture of *entB* mutant *K. pneumoniae*, supplemented with 4-fold serial dilutions of purified Ent or Gly-Ent (Salmochelin S4; EMC microcollections, Tuebingen, Germany) or culture supernatant, incubated overnight and plated for bacterial cfu. The dilution of culture supernatant (1∶640) where Lcn2-resistant growth matched that of Gly-Ent was used for comparisons.

### Quantification of neutrophils by flow cytometry

Antibody staining was performed at 4°C in 96-well plates using cells from 100 µL of nasal lavage fluid resuspended in PBS with 1% bovine serum albumin (BSA). For each mouse, cells were spun at 1500 rpm for 10 minutes, washed once with 200 µL PBS+BSA, centrifuged at 1500 rpm for 2 minutes, resuspended in the same volume and blocked for 10 minutes. Cells were spun as above, resuspended in 25 µL of a 1∶200 dilution of Fc Block (BD Pharmingen, San Diego, CA), and incubated an additional 10 minutes. Then, four-color staining was performed for 30 minutes by addition of 25 µL of an antibody mixture of fluorophore-conjugated Ly6G, CD11b, CD4, and CD45 antibodies (BD Pharmingen) at the following final concentrations: FITC 1∶200, PE 1∶300, PerCP 1∶100, APC 1∶300. Cells were washed twice, centrifuged and fixed by resuspension in 300 µL PBS+BSA with 0.5% paraformaldehyde.

For fluorophore compensation, mouse splenocytes were harvested. Spleens were homogenized by passage through a sterile screen, washed in 15 ml RPMI, and red blood cells were lysed by resuspension in 2 ml 0.83% ammonium chloride and incubation at room temp for 2 minutes. The suspension was neutralized by addition of RPMI, incubated an additional 3 minutes, and the supernatant was removed, centrifuged, washed in RPMI and resuspended in PBS+BSA. 200 µL aliquots were blocked for 10 minutes with PBS+BSA, and individually stained with FITC, PE, PerCP, or APC-conjugated anti-CD4 antibody. Flow cytometry data was collected using a BD FACSCaliber and analyzed using FlowJo software (Tree Star, Ashland, OR). For nasal lavage fluid, the entire sample was analyzed and normalized to events per ml. *Lcn2^+/+^* and *Lcn2^−/−^* littermates were compared pair-wise to account for potential variation in baseline neutrophil counts that may be caused by differences in the endogenous nasal flora.

### Cell culture and IL-8 release assay

The human type II pneumocyte A549 cell line (ATCC CCL-185) was propagated in minimal essential medium (Invitrogen, Carlsbad, CA) supplemented with 10% fetal bovine serum as described previously [8]. Near-confluent monolayers in 24-well plates were weaned from serum and antibiotics overnight. Then, A549 cells were stimulated with combinations of 50 µM purified siderophores (EMC microcollections, Tuebingen, Germany) or 25 µM lipocalin 2 overnight as indicated. Purified diglycosylated Ent (Salmochelin S4), which is the major product of IroB [41], or purified Ent was used. The next day, culture supernatants were collected and stored at −20°C or used immediately for IL-8 ELISA.

The BD OptEIA IL-8 ELISA assay (BD Biosciences, San Diego, CA) was performed according to the manufacturers instructions and detected using TMB substrate (Zymed/Invitrogen, Carlsbad, CA) and a Bio-Rad Model 680 microplate reader (Bio-Rad, Hercules, CA). As a positive control for IL-8 release, cells were stimulated with 100 pg/ml of IL-1β (Peprotech, Rocky Hill, NJ). A vehicle control was included containing the solvents for each reagent used. Cytotoxicity was evaluated by LDH measurement of supernatants using the Cytotoxicity Detection Kit Plus (Roche, Mannheim, Germany) according to the manufacturers directions.

### Statistical analysis

All statistical analysis was performed and graphs were generated using Prism 4 for Macintosh (GraphPad Software, Inc). For colonization data, non-parametric analysis of two groups was performed using the Mann-Whitney test and analysis of multiple groups was performed using the Kruskall-Wallis Test with Dunn's post-test. The presence or absence of splenic bacteria was analyzed by Fisher's Exact test. IL-8 ELISA data was analyzed by one-way ANOVA with Tukey's multiple comparison test. Cytotoxicity data was analyzed by one-sample t-test for significant increase above the control and one-way ANOVA with Tukey's multiple comparison test for differences between samples. For neutrophil quantification, non-parametric analysis of mouse pairs was performed using the Wilcoxon matched pair test.

### Nucleotide and protein accession numbers

Genbank nucleotide accession numbers for the *iroA* loci used for *iroB* sequence comparisons are NC_005249 (*K. pneumoniae* CG43 pLVPK), NC_003197 (*Salmonella* Typhimurium LT2), NC_004431 (*E. coli* CFT073), and NC_007946 (*E. coli* UTI89). Genbank accession number for the *iroBCDN* genes from χ7122 used for complementation is AF449498. The Genbank protein accession number for Lcn2 is NP_032517 for 24p3R is NP_067526.
